# Quality Control of RNA Preservation and Extraction from Paraffin-Embedded Tissue: Implications for RT-PCR and Microarray Analysis

**DOI:** 10.1371/journal.pone.0070714

**Published:** 2013-07-31

**Authors:** Karl Kashofer, Christian Viertler, Martin Pichler, Kurt Zatloukal

**Affiliations:** 1 Institute of Pathology, Medical University of Graz, Graz, Austria; 2 Division of Oncology, Medical University of Graz, Graz, Austria; Roswell Park Cancer Institute, United States of America

## Abstract

Analysis of RNA isolated from fixed and paraffin-embedded tissues is widely used in biomedical research and molecular pathological diagnostics. We have performed a comprehensive and systematic investigation of the impact of factors in the pre-analytical workflow, such as different fixatives, fixation time, RNA extraction method and storage of tissues in paraffin blocks, on several downstream reactions including complementary DNA (cDNA) synthesis, quantitative reverse transcription polymerase chain reaction (qRT-PCR) and microarray hybridization. We compared the effects of routine formalin fixation with the non-crosslinking, alcohol-based Tissue Tek Xpress Molecular Fixative (TTXMF, Sakura Finetek), and cryopreservation as gold standard for molecular analyses. Formalin fixation introduced major changes into microarray gene expression data and led to marked gene-to-gene variations in delta-ct values of qRT-PCR. We found that qRT-PCR efficiency and gene-to-gene variations were mainly attributed to differences in the efficiency of cDNA synthesis as the most sensitive step. These differences could not be reliably detected by quality assessment of total RNA isolated from formalin-fixed tissues by electrophoresis or spectrophotometry. Although RNA from TTXMF fixed samples was as fragmented as RNA from formalin fixed samples, much higher cDNA yield and lower ct-values were obtained in qRT-PCR underlining the negative impact of crosslinking by formalin. In order to better estimate the impact of pre-analytical procedures such as fixation on the reliability of downstream analysis, we applied a qRT-PCR-based assay using amplicons of different length and an assay measuring the efficiency of cDNA generation. Together these two assays allowed better quality assessment of RNA extracted from fixed and paraffin-embedded tissues and should be used to supplement quality scores derived from automated electrophoresis. A better standardization of the pre-analytical workflow, application of additional quality controls and detailed sample information would markedly improve the comparability and reliability of molecular studies based on formalin-fixed and paraffin-embedded tissue samples.

## Introduction

Quantitative measurement of messenger RNA (mRNA) levels by quantitative reverse transcription polymerase chain reaction (qRT-PCR), microarray techniques or the more recently established next-generation sequencing has become an essential tool for studying gene expression and has given new insights into a multitude of pathophysiological processes. Today, in routine pathology archives all over the world huge numbers of formalin-fixed paraffin-embedded (FFPE) tissue samples are stored and thereby provide an invaluable resource for investigating gene expression alterations in a broad spectrum of diseases. The universally used buffered 10% formalin fixative represents gold standard for histopathological diagnosis as it excellently preserves tissue morphology [1], [2]. However, fixation by formalin causes modifications of biomolecules, such as cross-linkage of nucleic acids with proteins, covalent modifications of RNA by the addition of multiple monomethylol moieties to the amino groups of bases or methylene bridging between neighboring bases and fragmentation of the RNA molecules [3], [4]. These alterations lead to suboptimal performance of RNA extracted from FFPE tissues in subsequent analysis. To overcome this problem, several groups have proposed specific additives to the standard fixative or the use of non-crosslinking organic fixatives to improve RNA yield and quality [5]–[11]. However, all these alternative fixation methods have their limitations, either with respect to preservation of the histopathological diagnostic features or in other applications, like immunohistochemistry [12].

In general, the quality of RNA derived from a tissue sample is affected by several parameters. These include the time between sample retrieval from the patient and fixation where ischemia and autolysis occurs [13], the duration and conditions of the actual fixation process, the paraffin embedding procedure, sample storage and finally the RNA extraction protocol [14]–[18]. These successive pre-analytical procedures influence the quality of the biomolecules derived from tissue samples and cannot be easily standardized between laboratories. As a consequence, RNA extracted from FFPE tissue often shows unpredictable variations in quantity and quality which may lead to varying and poorly reproducible gene expression data [19]. Several modifications of the reverse transcription reaction [20], [21] have been proposed to address these shortcomings, but it remains unclear if these modifications removed the variation between genes introduced by pre-analytical procedures. Hence, there is a need for a robust and easily applicable method for assessing the quality and quantity of RNA extracted from fixed and paraffin-embedded tissue to guarantee reliability of downstream analysis and diagnostic conclusions.

State-of-the-art RNA based analyses still rely mainly on fresh or cryopreserved samples, ensuring a much better preservation of biomolecules than fixation with formalin. In agarose gels or electropherograms intact total RNA extracted from cryopreserved tissue displays two distinct bands or peaks from the structural RNA of the eukaryotic 18 s and 28 s ribosome subunits (rRNA). The abundance, defined size and ratio of these two bands/peaks allows their use as surrogate markers of RNA fragmentation by calculating the ratio of the rRNA bands (28S:18S) or using the shape of the electropherogram to derive parameters, such as the RIN value (Agilent Technologies, Santa Clara, CA, USA). While these assays are well established for RNA from fresh or cryopreserved tissues, it is also known that the two distinct rRNA peaks are compromised in RNA extracted from tissues fixed in formalin or other denaturing fixatives [22]. It is furthermore unclear how well the fragment size distribution of the ribosomal RNA predicts the behavior of the messenger RNA (mRNA) subsequently used as source material for gene expression studies. Because rRNA is a structural component of the ribosome it could be extensively cross-linked to the amino acids of the ribosomal proteins leading to more extensive degradation of rRNA. In addition, the structural integrity does not necessarily correlate with chemical modifications and performance of RNA in enzymatic reactions. Several groups have already reported on the impact of formalin fixation on qRT-PCR [23]–[25]. Building on these previous reports we have now performed a comprehensive and comparative study of the impact of different pre-analytical steps on RNA fragmentation and performance in different analytical reactions. The goal was to identify the most critical pre-analytical steps that should also become the target for more accurate quality control for instance by using different assays presented in our study. Based on the assumption that the crosslinking of biomolecules is a major contributor to RNA degradation, we explored the detrimental effects of the routinely used crosslinking fixative formalin on RNA molecules in comparison with a non-crosslinking, alcohol-based fixative, by taking the Tissue Tek Xpress Molecluar fixative (TTXMF) as example and cryopreserved samples as reference for molecular analyses.

## Materials and Methods

### Ethics Statement

Sample donors provided written informed consent, and the study was approved by the Ethics Committee of the Medical University of Graz, Austria (reference number 20-066).

### Tissue Samples and Fixation

**Figure 1:**
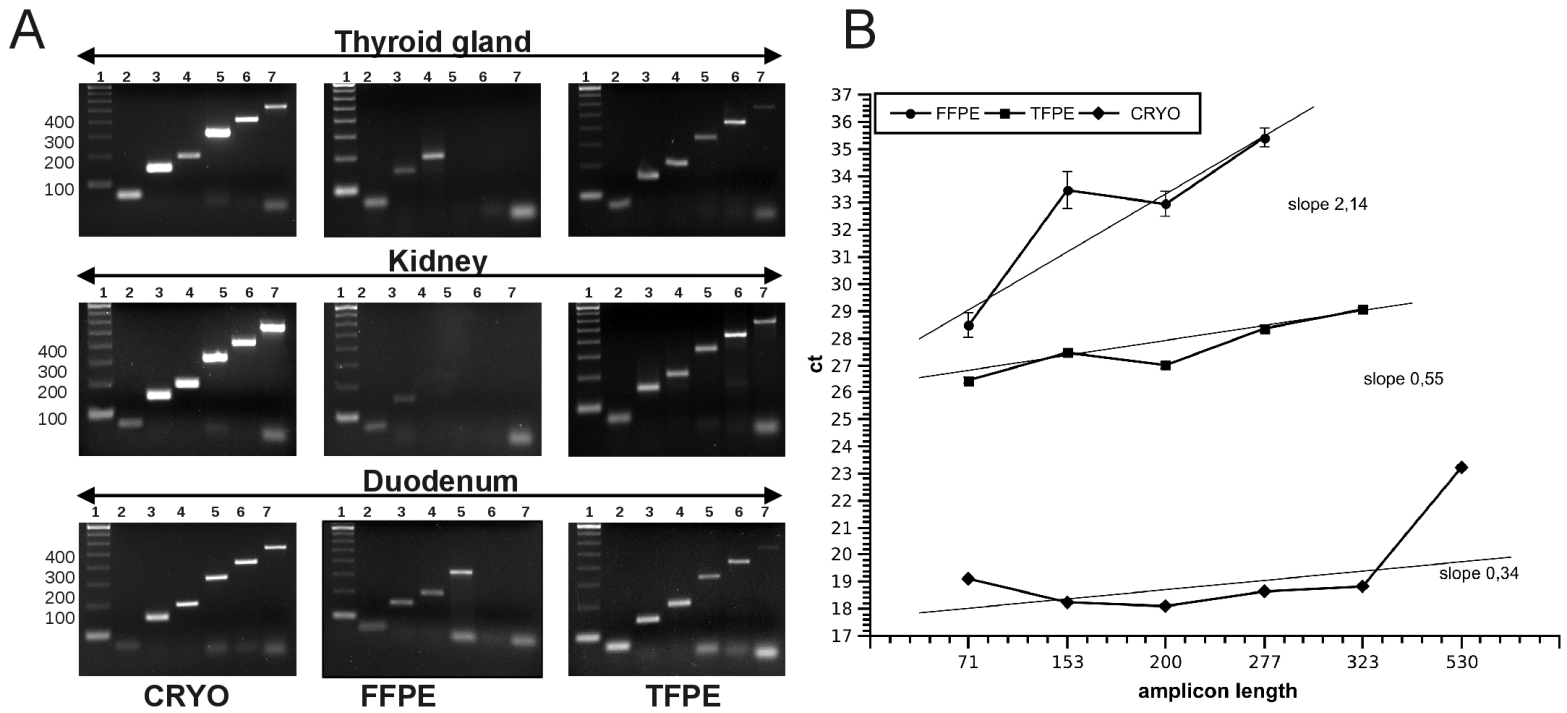
Assay for evaluation of RNA performance in qRT-PCR. qRT-of six amplicons of different length (71, 153, 200, 277, 323, 530 bp amplicon size) the gene GAPDH performed using cDNA derived from FFPE, TFPE and cryopreserved samples of different organs. (A) Gel images of GAPDH amplicons in three different CRYO, FFPE and TFPE tissues. All size fragments can be amplified from CRYO samples, most larger amplicons are missing in FFPE samples, all amplicons are present in TFPE samples but the 530 bp band is weak. (B) Direct comparison of ct values obtained from CRYO, FFPE and TFPE liver tissue. In FFPE samples the 323 and 530 bp bands could not be detected or were unspecific, and ct values of smaller amplicons were shifted by up to 9 cycles. The slope of the regression line through the data points was greater in FFPE (2.14) than in TFPE (0.55) and the cryopreserved (0.34) liver samples. Error bars depict standard deviation of PCR triplicates (too small to be visible in CRYO and TFPE samples).

Tissue samples for this study were obtained from patients undergoing routine surgical intervention at the University Hospital of Graz. Immediately after resection, tissue specimens were transferred to the pathology unit and were evaluated by an experienced pathologist. Corresponding samples were removed from each of the surgical specimens, divided into multiple aliquots of approximately 10×5×5 mm and processed in parallel either by (i) fixation in 10% buffered formalin and paraffin embedding (FFPE) or (ii) Tissue-Tek® Xpress® Molecular Fixative (TTXMF) followed by paraffin embedding (TFPE) according to manufacturer’s instructions or (iii) snap-frozen in methylbutane pre-cooled by liquid nitrogen and stored in liquid nitrogen (CRYO). In total 99 samples from 28 patients were procured. Figure 1 shows a summary of RIN values and GAPDH quality control assay results comparing diverse malignant and non-malignant tissue samples from different organs (e.g. stomach, duodenum, colon, rectum, liver, spleen, and kidney) and patients. To reduce biological variability and facilitate a direct comparison of the pre-analytical effects we performed subsequent experiments on multiple aliquots of the same specimen to highlight the differences introduced by sample processing. Several of the experiments have been performed on liver tissue because liver is an organ with a relatively homogeneous cellular composition allowing the generation of several aliquots for parallel investigation of different pre-analytical factors. Hematoxylin and eosin stainings of different FFPE and TFPE samples used in this study (thyroid gland, kidney, liver) are shown in Figure S1.

### RNA Extraction

Total RNA was extracted from cryopreserved tissue using Trizol (Invitrogen Life Technologies), following the manufacturer’s protocol. Briefly, 80–120 mg of tissue sections were added to 1 ml Trizol, homogenized with an Ultra-Turrax and incubated at room temperature for 5 minutes (min). After chloroform extraction and precipitation with isopropanol, RNA was washed twice with 75% ethanol, and finally the RNA pellet was dissolved in 15 µl of RNase-free water.

Total RNA from paraffin-embedded samples (FFPE and TFPE) was extracted using the extraction protocol described by Specht et al [26]. Briefly, unstained 5 µm thick sections were prepared, using an RNase-free microtome and placed in an RNase-free tube. Paraffin was removed by extracting twice with xylene for 10 min. This step was followed by rehydration through subsequent 5 min washes with 100%, 90% and 70% ethanol that was diluted with RNase-free water. After the final washing step, the pellet was dried, resuspended in 200 µl of RNA lysis buffer (containing 10 mM/L Tris-HCl, pH 8.0; 0.1 mM/L EDTA, pH 8.0; 2% SDS, pH 7.3; 500 µg/ml proteinase K) and incubated at 60°C overnight. Then, the RNA was purified by phenol/chloroform extraction and precipitated with an equal volume of isopropanol and 0.5 µl of 20 mg/ml glycogen at −20°C. After centrifugation (30 min/13000 rpm/4°C), the RNA pellet was washed with 70% ethanol and finally dissolved in 15 µl RNase-free water. Alternatively, RNA extraction was performed using the RNeasy FFPE Kit (Qiagen, Hilden, Germany) according to manufacturer’s instructions. RNA concentration and purity were determined using a NanoDrop ND-1000 spectrophotometer (Thermo Fisher Scientific, USA). Electropherograms were obtained using an Agilent 2100 Bioanalyzer platform (Agilent Technologies, USA) with an Agilent RNA 6000 Nano Kit; Agilent 2100 Expert software version B.02.03.SI307 was used to calculate the RNA integrity number (RIN).

### RNA Purification and Complementary DNA (cDNA) Synthesis

Where indicated, the total RNA obtained by Trizol extraction was purified by processing with RNeasy Mini Kit (Qiagen) or RNeasy miRNA Mini Kit (Qiagen) according to manufactureŕs instructions. After extraction and purification, the RNA yield and purity was determined by measuring absorbance at 260 nm/280 nm on a Nanodrop spectrophotometer (Thermo Fisher Scientific). The Agilent 2100 Bioanalyzer (Agilent Technologies) was used to profile and compare RNA fragment length from the three different preservation methods with the RNA 6000 LabChip kit (Agilent Technologies). Electropherograms were analysed with Agilent 2100 expert software. To generate cDNA for the experiments shown in Figure 1, RNA was reversely transcribed using Superscript II reverse transcriptase kit (Invitrogen), according to manufactureŕs instructions. Reactions were performed in a final volume of 20 µl using the buffer provided by the manufacturer, supplemented with 12 mmol/L dNTPs, 40 U/µl of RNase inhibitor (Invitrogen), 0.3 µl (5 nM) of random hexamers and 1 µg of total RNA. Reverse transcription was performed in a thermocycler (GeneAmp 9700, Applied Biosystems) under the following reaction conditions: 65°C for 5 min, 42°C for 90 min and 90°C for 15 min. cDNA for all other experiments was generated by reverse transcription with the High Capacity Archive cDNA generation kit (#4322171, Applied Biosystems) according to manufactureŕs instructions.

### qRT-PCR Assay for Assessment of RNA Quality

We established an assay based on the housekeeping gene glyceraldehyde-3-phosphate dehydrogenase (GAPDH), a gene that is expressed in most tissues at a relatively constant level. Seven amplicons with different lengths (71, 153, 200, 277, 323, 413 and 530 bp) were designed with Primer3 software. One common GAPDH forward primer and six different GAPDH reverse primers were designed: common forward: 5′-CCACATCGCTCAGACACCAT-3′; 71 bp: 5′-GTAAACCATGTAGTTGAGGTC-3′; 153 bp: 5′-ACCAGGCGCCCAATACG-3′; 200 bp: 5′-TTGACGGTGCCATGGAATTT-3′; 277 bp: 5′-ACTTGATTTTGGAGGGATCT; 323 bp: 5′-AAGACGCCAGTGGACTCC A; 413 bp: 5′-TGACGAACATGGGGGCATCA-3′; 530 bp: 5′-ACGATACCAAAGTTGTCATG-3′. The amplicon with a length of 413 bp was no longer used after optimization of the assay. Endpoint PCR assays were performed in 30 µl final volume containing PCR buffer (Invitrogen), 2 mM of the DNA polymerization mix (Pharmacia, 20 mM), AmpliTaq DNA Polymerase (QIAGEN; 5 Units/µl) and 10 pmol/µl of each specific primer. PCR was initiated with a 30-second step at 94°C, followed by 40 cycles of denaturation at 94°C for 30 seconds, primer annealing step at 60°C for 30 seconds and a 30-second extension at 72°C. Aliquots (14 µl) of PCR product were loaded on a 2% SeaKem ME Agarosegel (Cambrex) and separated in 1 x TAE running buffer at 80 V for 30 min in an electrophoresis chamber (Biorad). Ethidium bromide stained DNA bands were visualized by illumination with ultraviolet light (E.A.S.Y RH/UVT-28 MP Herolab).

Additionally, a real-time qRT-PCR protocol for assessment of RNA quality and quantity was developed on the ABI PRISM® 7900HT Sequence Detection System (Applied Biosystems). qRT-PCR was conducted in 96-well optical reaction plates using a final volume of 25 µl. Optimum reaction conditions were obtained with 15 µl of 2× SYBR^®^ Green PCR MasterMix (Applied Biosystems), 1 pmol specific forward primer and 1 pmol specific reverse primer. Finally, 25 ng template cDNA was added to the reaction mixture and amplifications were performed using the standard HT7900 program. All samples were amplified in triplicates, and the mean cycle threshold value (ct) was calculated for further analysis. After amplification melting curve analysis was used to exclude unspecific products and primer dimers and selected PCR products were checked on 2% agarose gels.

### Microarray Analysis

20 µg of the RNA preparations extracted from a FFPE, TFPE and cryopreserved human liver adenoma were reverse transcribed using the Chemiluminescent RT Labeling Kit (#4359029) and hybridized to the Human Genome Survey Microarray (#4337467) with the Chemiluminescence Detection Kit (#4342142, all by Applied Biosystems). Microarrays were analyzed on the 1700 Chemiluminescent Microarray Analyzer and data was exported with AB 1700 Plate Reader 1.2.2. software into Genespring v7.3 (Agilent Technologies, Waldbronn, Germany) for background correction and quantile normalization across all arrays in the experiment. Pearson correlation coefficients were calculated from all data points of the respective analyses to show statistical correlation of the results obtained from different samples.

### TaqMan Gene Expression Assays and TaqMan Array Gene Signature Plate

2 µg of RNA from TFPE, FFPE, and cryopreserved human liver samples were transcribed into cDNA using the High-Capacity cDNA Reverse Transcription Kit (Applied Biosystems). Applied Biosystems Gene expression assays for GAPDH (Hs99999905_m1), beta-2-microglobin (B2M, 4310886E-0211007), CD14 molecule (CD14, Hs00169122), alpha-fetoprotein (AFP, Hs00173490_m1), interleukin 7 (IL7, Hs00174202_m1) and tumor necrosis factor receptor superfamily, member 19 (TNFRSF19, Hs00218634_m1) were used according to manufacturer’s instructions. For analysis of cancer gene signature 10 µl of TaqMan Gene Expression Master Mix was added to 10 µl of cDNA leading to a final concentration of 20 ng per 20 µl reaction. Samples were analyzed on the AB 7900 Real-Time PCR System using the preconfigured TaqMan Array 96-Well Plate Human Molecular Mechanisms of Cancer including 92 pathway-associated genes and 4 endogenous control genes. Results were analyzed using SDS 2.3 software (all by Applied Biosystems) and Pearson Correlation coefficient was calculated from different sample types and technical replicates in comparison to the corresponding cryo reference.

### Ribosomal RNA Assay

Primer sets for the amplification of the 5 s, 18 s and 28 s ribosomal RNA were designed. (5 s: 5′-CTAGCTGCGAGAATTAATGTG-3′, 5′-GAAGTGTCGATGATCAATGTG-3′, 51 bp; 18 s: 5′-TTGCGTTGATTAAGTCCCT-3′, 5′-TCACTAAACCATCCAATCGG-3′, 68 bp; 28 s: 5′-CCTCACGATCCTTCTGAC-3′, 5′-CCACAAGCCAGTTATCCC-3′, 76 bp). RNA was extracted from tissue samples as described previously and 2 µg of RNA were reversely transcribed with the High Capacity Archival cDNA generation kit (Applied Biosystems). qRT-PCR was performed with the SYBR^®^ Green PCR MasterMix (Applied Biosystems) using the default temperature profile. Due to the abundance of ribosomal RNA species in total RNA preparations only 5 ng of cDNA were used in each qRT-PCR.

### Measurement of Reverse Transcription (RT) Efficiency

RNA was extracted from a FFPE, TFPE and cryopreserved human liver sample as described before. RNA was then digested with DNAse to remove contaminating genomic DNA. cDNA was generated from different amounts of RNA (0 µg, 0.125 µg, 0.25 µg, 0.5 µg, 1 µg) by using the Applied Biosystems High Capacity Archival cDNA generation kit. After the cDNA reaction the template RNA was removed by alkaline hydrolysis and the remaining cDNA concentration was determined using RiboGreen (Invitrogen) as described previously [27]. Briefly, cDNA was generated according to manufacturer’s recommendation in a volume of 20 µl. A sample without reverse transcriptase (no-RT) was taken from the reverse transcription reaction mix before addition of the enzyme and incubation at 37°C for 2 hours (hrs). EDTA and NaOH were added to 5 µl of the no-RT sample and to 5 µl of the cDNA sample to obtain a final concentration of 1 mM EDTA and 100 mM NaOH in a volume of 20 µl. The samples were incubated at 70°C for 20 min to hydrolyze all RNA molecules and then the pH was neutralized by addition of 6 µl of 0.5 M Tris-HCl pH 6.4. 25 µl of these reactions were then incubated with 1 µl Ribogreen RNA Quantitation Reagent (R-11491, Molecular Probes) in a volume of 200 µl and transferred to microplate wells. Samples were excited at 485 nm and the fluorescence emission intensity was measured at 530 nm using a Synergy HT Multi-Mode Microplate Reader (Biotek, Germany). The difference in fluorescence of the no-RT to the cDNA sample corresponds to the amount of cDNA generated in the reverse transcription reaction.

## Results

Gene expression analysis from formalin-fixed tissue samples has several inherent limitations. It is well known that ct-values generated by qRT-PCR from RNA extracted from formalin-fixed and paraffin-embedded (FFPE) are higher than from cryopreserved tissue (CRYO). To accurately measure the PCR-ability of RNA from CRYO and FFPE tissues we designed an assay for quantitative assessment of PCR efficiency for different amplicon lengths (71, 153, 200, 277, 323 and 530 bp) from the housekeeping gene GAPDH. When performed as a standard end-point PCR with RNA extracted from cryopreserved human thyroid gland, kidney and duodenum, the amplification of all fragment sizes was of largely similar efficiency (Fig. 1A, left column). Using RNA extracted from FFPE tissue only smaller PCR products were detectable (Fig. 1A, center column). Longer PCR products were obtained using RNA extracted from TFPE tissue with amplification of all fragments present in kidney samples, and only the largest fragment (530 bp) missing in thyroid gland and duodenum samples (Fig. 1A, right column). Performing this PCR assay as real-time reactions on the Applied Biosystems TaqMan quantitative PCR platform allowed comparing the ct values for the different amplicons of the GAPDH mRNA between RNA samples (Fig. 1B). The ct values were between 18 and 20 for the first 5 amplicons, and 23 for the largest amplicon of 530 bp in cryopreserved tissue. In contrast, for RNA from FFPE samples even the smallest amplicon had a ct of 28 and subsequent amplicons showed even higher ct values. Lower ct values were obtained from RNA of TFPE samples, with 26 cycles for the smallest amplicon and up to 28 for the 323 bp amplicon. 323 bp or 530 bp sized fragments could not be detected in FFPE samples, while in TFPE samples only the 530 bp amplicon was absent. Comparing the slope of the regression line drawn through the data-points of the different preparations revealed a very high slope (2.14) in the data-points of the FFPE samples and a much lower slope in the cryopreserved (0.34) or TFPE samples (0.55), (Fig. 1B) demonstrating the impact of RNA fragmentation on the efficiency of PCR in relation to amplicon length.

**Figure 2:**
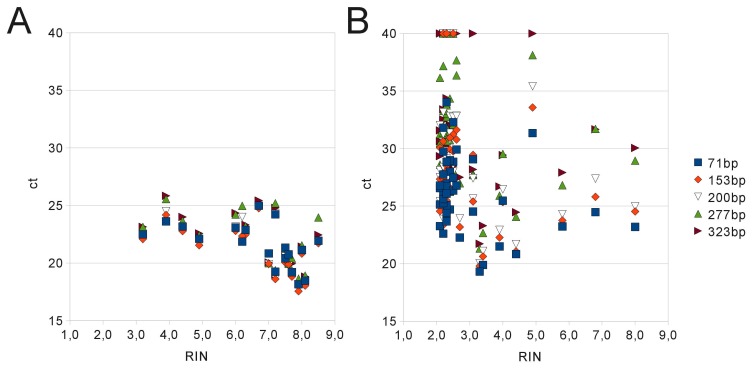
Comparison of RIN values and qRT-PCR results of different FFPE and cryopreserved samples. RIN and ct-values generated by qRT-PCR of RNA extracted from 65 different samples of 15 patients are shown. Corresponding aliquots of different tissue types were (A) cryopreserved or (B) fixed in formalin and embedded in paraffin. The majority of cryopreserved samples have RIN values above 5 while the majority of FFPE samples show low RIN values between 2 and 3. 11 FFPE samples showed high RIN values above 3 and up to 8 but ct values of the qRT-PCR assays of these samples were not substantially lower than in the other FFPE samples. In CRYO samples the Pearson correlation factor between RIN and ct values is −0.5 to −0.6, in FFPE samples the correlation factor is −0.25 to −0.28 depending on amplicon length. The difference in ct values obtained from CRYO or FFPE samples is statistically significant (p<0.01). PCR was performed in triplicates and median ct is shown. Samples not amplifying or yielding unspecific products were set to a ct of 40 (but omitted from statistical analysis).

This observations in four different tissue types was further investigated in a larger cohort of FFPE tissue samples (n = 46) fixed for varying time periods and corresponding CRYO tissue samples (n = 19) used as reference (Fig. 2). The difference of ct-values obtained in these groups was statistically significant (p<0.01) and the influence of amplicon length on qRT-PCR efficiency was consistantly more pronounced in FFPE samples than in CRYO samples. Additionally, ct values obtained from cryopreserved samples correlated with RIN values generated by the Agilent Bioanalyzer with a pearson correlation coefficient of −0.5 and −0.6 depending on amplicon size. In contrast, this correlation was much lower (−0.25 to −0.28) in FFPE fixed tissues where the majority of samples displayed RIN values between 2 and 3, while ct-values showed a wide range from 18 to 36 depending on amplicon length (Fig. 2B). Notably, 11 FFPE samples showed high RIN values above 3 and up to 8 but ct values of the qRT-PCR assays of these samples were not substantially lower than in the other FFPE samples. These results indicate major differences in qRT-PCR efficiency between various FFPE samples which is not properly detected by using RIN as quality control parameter.

**Figure 3:**
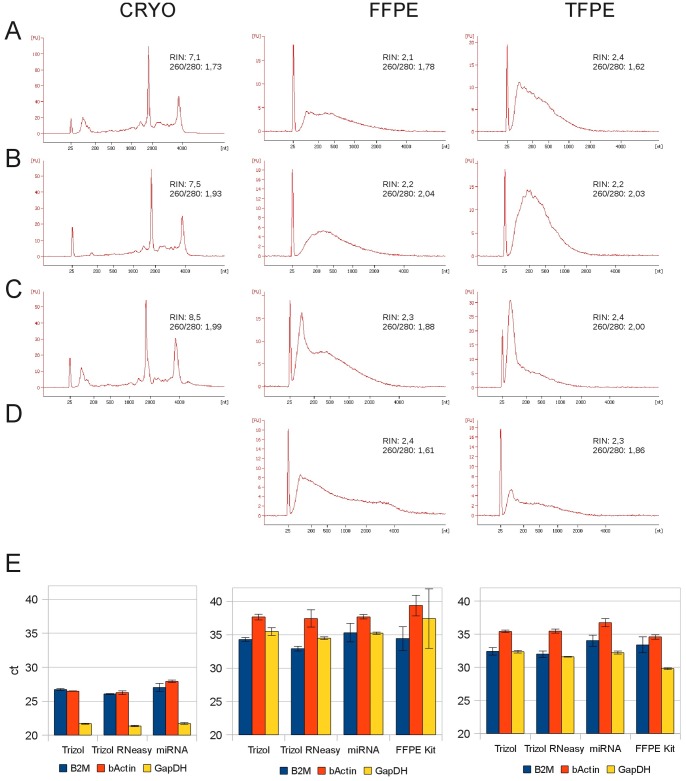
Fixation has strong influence on RNA fragment size and impairs qRT-PCR results. Electropherograms show different RNA molecule size distribution in cryopreserved and FFPE/TFPE liver tissue samples. (A) In contrast to CRYO, ribosomal RNA peaks are absent and fragmentation of RNA has occurred in all fixed tissues. (B) RNA cleanup by Qiagen RNeasy Kit removes small (<200 bp) RNA molecules. (C) RNA extraction using the RNeasy miRNA kit (Qiagen) enriches for small RNA fragments. (D) Extraction with RNeasy FFPE kit (Qiagen) does not change the overall size distribution of fragments but also enriches small molecules. Purification on column based systems (B–D) yields higher 260/280 ratio indicating less contamination of RNA. (E) Fixation has strong impact on the raw ct values and standard deviation of triplicates in qRT-PCR of three different housekeeping genes in cryopreserved, FFPE and TFPE tissue. Note that fixation markedly changes the ratio of ß2 microglobin (B2M) to GAPDH and this effect is further influenced by RNA isolation method used.

Differences in the efficiency of qRT-PCR are often attributed to impurities or contaminations in RNA samples. To elucidate the impact of different extraction and purification protocols on qRT-PCR efficiency we performed RNA extractions from a human liver adenoma case with homogeneous morphology in mirrored FFPE, TFPE and cryopreserved aliquots (Figure S1C). Extractions were performed in parallel by using several well established protocols in order to compare the quality of the resulting RNA. RNA can be extracted from tissues either by Trizol Reagent (Invitrogen, Carlsbad, USA), or by commercially available kits which utilize columns containing silica matrices or membranes that can bind nucleic acids in the presence of high salt. Trizol extraction of RNA is a very common, cost-effective and reliable method for RNA extraction from fresh and cryopreserved tissue, and can also be used for fixed tissue if proteinase K digestion is performed before extraction [28]. The electropherogram of RNA extracted with the Trizol method from cryopreserved tissue (CRYO) showed a distinct curve with two clear peaks for the ribosomal RNA (rRNA) and a RIN value of 7.1 which indicates RNA of high integrity (Fig. 3A, top panel). RNA extracted from the same tissue fixed in formalin or TTXMF followed by paraffin embedding (FFPE, TFPE) showed no distinct rRNA peaks in the electropherogram and a RIN value of 2.1 and 2.4, respectively (Fig. 3A, top panel). After extraction of RNA by Trizol it is often advised to remove contaminants from the RNA preparation by processing it with a column based RNA extraction/purification kit like the RNeasy Kit (Qiagen). As shown in Fig. 3B, this cleanup of the RNA samples removed contaminants and RNA fragments smaller than the cutoff of the columns which is at approximately 100 bp for the RNeasy kit leading to a higher 260/280 ratio. We speculated that the rRNA and mRNA of fixed samples was fragmented into small molecules and that these small fragments might be lost during the RNA extraction. To avoid this loss we also performed the extraction from these tissues with the RNeasy miRNA kit (Qiagen) which has no size cutoff and should be better suited to recover smaller fragments present in RNA from fixed samples. Indeed, as shown in Fig. 3C, RNA purified with the miRNA kit is enriched in small fragments which may be degradation products of rRNA and mRNA. We also tested the Qiagen RNeasy FFPE kit which was marketed for direct extraction of RNA from FFPE tissue. The extraction was done from both formalin and TTXMF fixed samples and the electropherograms of the RNA showed that this kit also enriched smaller RNA fragments (Fig. 3D). With all the different extraction methods the RIN values of the RNA extracted from cryopreserved tissue were above 7 indicating unfragmented RNA of good quality. RNA extracted from fixed and paraffin-embedded samples showed RIN values in the range of 2.1 to 2.4 suggesting a high degree of RNA fragmentation and low RNA quality regardless of the extraction protocol used.

A very common application after RNA extraction is the determination of gene expression values by qRT-PCR. We have determined the abundance of individual mRNA targets in a variety of RNA samples generated from cryopreserved, FFPE and TFPE tissues to analyze the suitability of the individual preparations for qRT-PCR. We obtained ct values from all samples using primers for B2M, beta-actin (ACTB) and GAPDH using a Sybr-green based assay on the TaqMan platform (Applied Biosystems, CA, USA). The ct values of the qRT-PCR amplifications were 25–27 for B2M and ACTB, and 22 for GAPDH in preparations from cryopreserved tissues (Fig. 3E). In contrast, in samples generated from FFPE tissues the ct values were much higher, between 32 and 37 and also had a higher standard deviation. In addition to the significantly higher ct we have observed a change in the relative ratio of gene expression between these common housekeeping genes. In the qRT-PCR analyses performed with RNA from TFPE we observed a similar, albeit not as large rise of ct values while the standard deviations were generally smaller than in FFPE samples. However, the change in the relative ratios between the housekeeping genes was also present in these samples (Fig. 3E).

**Figure 4:**
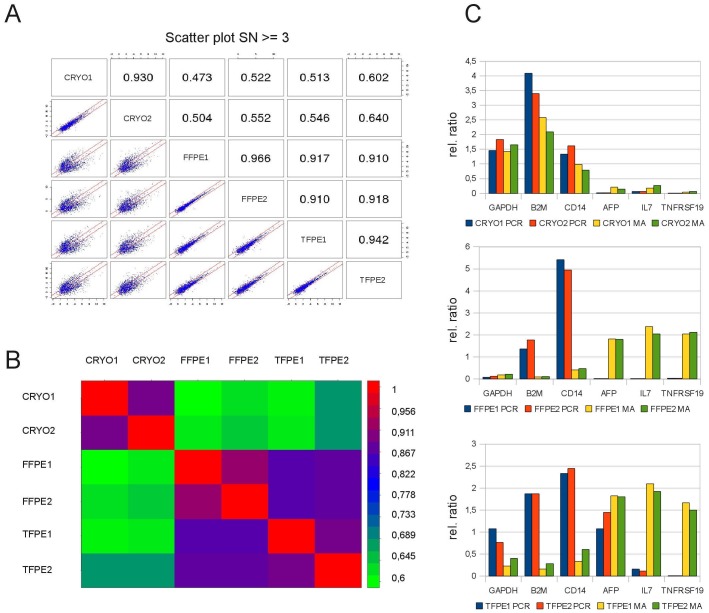
Fixation impairs gene expression profiles as determined by 5′IVT microarrays. (A) Scatter plots and Pearson correlation highlight the statistical distance of data generated from cryopreserved versus fixed samples by microarray analysis. (B) Signal correlation of cryopreserved samples with fixed samples is low (green) while fixed samples show high correlation (blue-red) within duplicates of one fixation method and also between FFPE and TFPE samples. (C, top panel) Comparison of qRT-PCR (PCR) results with microarray data (MA) measured in replicates for 6 genes reveals that similar results for qRT-PCR and microarray are only obtained in cryopreserved samples. (C, lower panels) In FFPE and TFPE samples the results of qRT-PCR are markedly different from the results generated by microarray analysis. Panels depict data normalized to the mean of all measurements of a group. Data was generated from a human liver tissue sample preserved by the indicated fixatives, two aliquots each. Each aliquot was analyzed by 3′-IVT microarray and qRT-PCR (in triplicates).

Gene expression analysis of RNA samples is not only performed by qRT-PCR, especially in biomedical research microarray analysis is used to generate gene expression data on a genome-wide level. Therefore we have additionally analyzed RNA from these liver adenoma samples using the Applied Biosystems Whole Genome 2.0 microarray platform to elucidate how RNA degradation and modification by fixation influences data generated with microarray techniques, and to evaluate whether there was a difference in the extent by which the mRNA of individual genes is affected. Analysis of the data generated with this platform allowed visualization of the correlation between gene expression values in technical replicates and between different fixation techniques, highlighting changes in gene expression data that are introduced solely by the fixation method and the subsequent embedding process (Fig. 4A). Pearson correlation of duplicate microarray experiments from cryopreserved samples was high (0.93), and also correlation in between the FFPE and the TFPE duplicates was high (0.97 and 0.94, respectively). Good correlation was also found between these two fixation methods ranging from 0.91 to 0.92. However, correlation between cryopreserved samples and fixed samples was generally poor, ranging from 0.47 to 0.64 (Fig. 4A and B).

To elucidate whether these differences in gene expression were specific to the microarray platform or were intrinsic properties of the different RNAs, we validated the expression of several genes by qRT-PCR. Six genes (GAPDH, B2M, CD14, AFP, IL7 and TNFRSF19) were chosen as they displayed large differences in expression measured by microarray between different fixation methods. GAPDH, B2M and CD14 were expressed higher than AFP, IL7 and TNFRSF19 in the cryopreserved tissue by microarray analysis (Fig. 4C, top panel, green and yellow bars, labeled MA). Validation by qRT-PCR utilizing TaqMan Gene Expression Assays confirmed this pattern of expression (Fig. 4C, top panel, blue and red bars, labeled PCR) establishing a high correlation of gene expression results obtained by microarray or qRT-PCR in cryopreserved samples.

In contrast, the expression values obtained by microarray technology from FFPE tissues were markedly different to expression values obtained from cryopreserved tissue, with low GAPDH, B2M and CD14 expression and high AFP, IL7 and TNFRSF19 expression (Fig. 4C, center panel, green and yellow bars, MA). Measuring the gene expression by qRT-PCR yielded results closer to the cryopreserved samples, which could be considered reflecting the “true” biological situation, with B2M and CD14 expressed higher than AFP, IL7 and TNFRSF19. RNA isolated from TFPE samples also showed contradictory results (Fig. 4C, lower panel) in the microarray experiment and again validation with qRT-PCR established gene expression values more similar to the cryopreserved tissue. This indicates that qRT-PCR is more robust than the microarray technique for measuring gene expression in RNA extracted from fixed tissues. However, some values like GAPDH in FFPE samples, and AFP in TFPE samples still differed significantly from the results obtained from the cryopreserved tissue.

**Figure 5:**
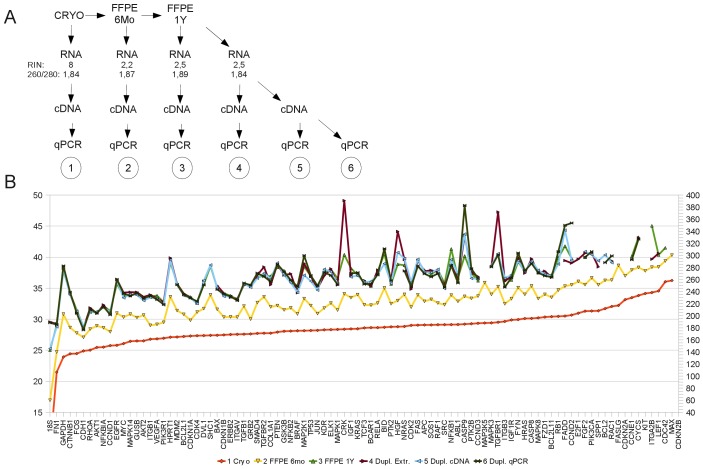
Fixation and storage introduces major gene-to-gene variations in qRT-PCR. Aliquots of a human liver sample were cryopreserved or fixed in formalin and paraffin embedded. (A) RNA was extracted from samples at different timepoints including technical replicates. (B) Comparison of qRT-PCR data for 92 genes from cryopreserved and FFPE human liver samples reveals an average difference of the ct values ranging from 4 cycles (6 months) to 8 cycles (1 year) increasing with storage time at room temperature. Extraction from the FFPE sample which had been stored for one year was done in duplicates. cDNA generation was performed in duplicates from the same RNA, and qRT-PCR was performed twice from the same cDNA. Data was generated with the TaqMan “Human Molecular Mechanisms of Cancer” assay, individual ct values are shown.

To assess the impact of fixation and subsequent storage on the quality of data generated by qRT-PCR we have analyzed RNA from cryopreserved and FFPE tissues stored for different periods of time using the Applied Biosystems TaqMan „Human molecular Mechanisms of Cancer“ assay. This assay comprised qRT-PCR analysis for 92 cancer pathway associated genes and four assays for endogenous control genes. We analyzed gene expression of corresponding aliquots from two different human livers (one case shown in Fig. 5), a colon cancer case and a leiomyosarcoma case which were cryopreserved or formalin-fixed and paraffin-embedded in parallel. In one of the non-malignant liver cases, we additionally analyzed different storage time points of 6 months and one year which reflects routine diagnostic workflows (Fig. 5). RNA was extracted twice from the one year FFPE sample to assess the impact of the extraction, cDNA generation of this RNA was done in duplicates to see variability introduced by fixation on reverse transcription, and the whole assay was performed twice from one cDNA sample as a technical replicate establishing the analytical variability of this qRT-PCR platform (Fig. 5A).

The ct values of the cryopreserved sample were sorted from lowest to highest resulting in a line with most ct values in a range of 25 to 35 cycles (Fig. 5B, sample 1, orange line). It is clearly visible that the ct values of the cryopreserved sample were the lowest among all samples. Formalin fixation, paraffin embedding and 6 months of storage increased the ct values of all genes by approximately 4 cycles (Fig. 5B, sample 2, yellow line). Furthermore, we observed major gene-to-gene variations introduced by formalin fixation and paraffin embedding already at this early time-point, indicated by a more pronounced rise in the ct values of single genes leading to spikes in the line and yielding a Pearson correlation of 0,941 when compared to CRYO. After one year of storage the average ct was 8 cycles higher than in the cryopreserved sample and gene-to-gene variation was even more pronounced (Fig. 5B, sample 3, light green line), reflected in a Pearson correlation of 0,7944 compared to CRYO. Additionally we observed that genes which showed the most pronounced variation in the 6 month sample were the same genes (e.g. AKT1, BID, CDC42, Map3K5 or PTK2) that showed even stronger variation in the 1 year storage sample. In contrast to the strong alteration of the gene expression values due to fixation, embedding and storage, the error introduced by RNA extraction, cDNA generation or the PCR was rather small, indicated by the close match of the results obtained from these four samples (samples 3–6, light green, red, blue, dark green).

**Figure 6:**
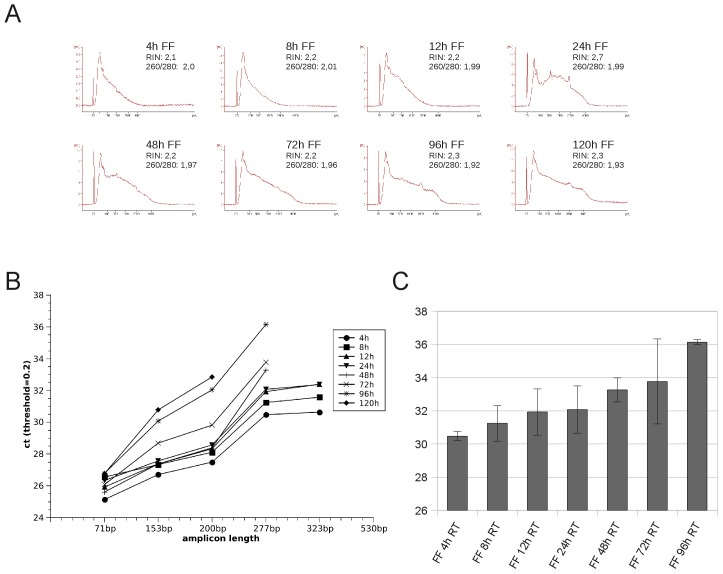
Formalin fixation impairs RNA integrity in a time dependent manner. Multiple aliquots of a human liver sample were fixed for different time periods ranging from 4 hr to 120 hr in formalin before paraffin embedding. (A) Similar RIN values were obtained for all fixation timepoints ranging from 2.1 to 2.7. (B) qRT-PCR amplification of fragments of different length in the GAPDH assay reveals a rise in ct values of all amplicons correlating with the fixation time. Prolonged fixation induces a steeper slope of the generated curves indicating increased fragmentation and makes the amplification of fragments longer than 277 bp impossible. (C) The ct values obtained for the 277 bp amplicon are correlating with the fixation time. qRT-PCR was performed in triplicates. Graphs depict median ct of triplicate qRT-PCR analyses, error bars depict standard deviation.

Formalin has a specific penetration and fixation rate depending on tissue composition which influences the time needed to properly preserve the whole tissue specimen. However, prolonged fixation time negatively influences tissue section quality, accessibility of antigens in immunohistochemistry and also RNA preservation In routine diagnostic laboratories tissues are usually fixed overnight in formalin at room temperature. Shorter fixation does not properly preserve the tissue morphology for subsequent analysis [29] and overfixation is known to impair antigen accessibility in immunohistochemical assays [30]. To evaluate the impact of fixation time we performed the GAPDH amplicon length assay mentioned above on multiple aliquots of another human liver sample fixed for periods of 4 hrs and up to 120 hrs in formalin before paraffin embedding and compared it with electropherograms and RIN values obtained by the Agilent Bioanalyzer. RIN values showed no discernible differences in the samples used, ranging from 2.2 to 2.7 with 260/280 ratios between 2.01 and 1.93 (Fig. 6A). As shown in Fig. 6B the shortest formalin fixation period (4 hrs) resulted in ct values which were 2 to 6 cycles lower than for the longest fixation period (120 hrs) depending on amplicon length. The longest (530 bp) fragment could not be amplified in any of the FFPE samples while the 323 bp fragment could only be amplified in fixations up to 24 hrs. If fixation time was further increased no fragments longer than 200 bp could be amplified and the slope of the regression line of the ct values was increased (Fig. 6B, left panel). The correlation between fixation time and RNA quality was clearly demonstrated by the ct values of primers amplifying the 277 bp fragment. This primer set showed successful amplification in most samples and ct values correlated with the RNA degradation introduced by the formalin fixation time (Fig. 6B, right panel).

**Figure 7:**
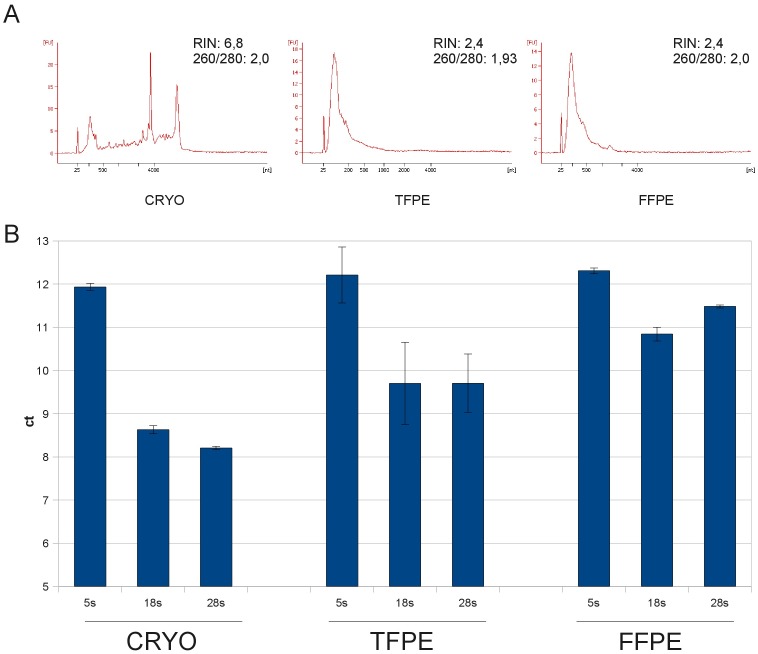
Fixation introduces a bias towards small RNA species in qRT-PCR. (A) Small RNA fragments like 5 s rRNA (∼120 bp) are present in cryopreserved (left) and fixed (right) samples of human liver. (B) Longer RNA fragments including 18 s (1,9 kb) and 28 s (5 kb) show an increase of 2 or 3 cycles in TFPE and FFPE samples respectively, revealing a stronger impact of fixation on larger RNA fragments. Bar graphs depict median of qRT-PCR triplicates and standard deviation.

The disappearance of the peaks of the 18 s and 28 s ribosomal RNA species is a well-known consequence of the fixation and paraffin embedding process. However, the implications of this disappearance are not clear. Therefore we analyzed FFPE, TFPE and cryopreserved samples of two different human liver cases. We assumed that the long rRNA molecules could be broken down into small fragments which are successfully extracted and still present in the mass of small fragments visible in electropherograms of RNA extracted from fixed and paraffin-embedded tissues (Fig. 7A). On the other hand it is also reasonable to assume that extensive crosslinking of long rRNA species to the protein structure of the ribosomes prevents their extraction by standard methods leading to a more pronounced loss of these molecules in the RNA preparation as compared to other RNA species with less tight protein association. To test these hypotheses we designed primers for the three rRNA species 5 s, 18 s and 28 s with very short amplicon lengths (50–70 bp) to determine whether fixation leads to a selective loss of rRNA molecules. Ct values from these three RNA species were 12, 8.5 and 8.2, respectively, in RNA isolated from cryopreserved tissue (Fig. 7B, left panel). When the same qRT-PCR were performed in RNA from FFPE samples the ct value of the smallest molecule, the 5 s rRNA, did not change significantly, whereas the ct values of the larger molecules did raise by approximately 3 cycles in the fixed samples indicating a substantial change in the relative ratio of these highly abundant RNA species. In RNA extracted from TFPE samples this effect was also present. However, in TFPE samples only a rise of 1.5 cycles was observed as compared to cryopreserved tissue (Fig. 7B). We conclude that although both large ribosomal RNA peaks disappear in the electropherograms of RNA from fixed tissue, most of the rRNA is still present and contained in the mass of small fragments visible in electropherograms of RNA extracted from fixed samples.

**Figure 8:**
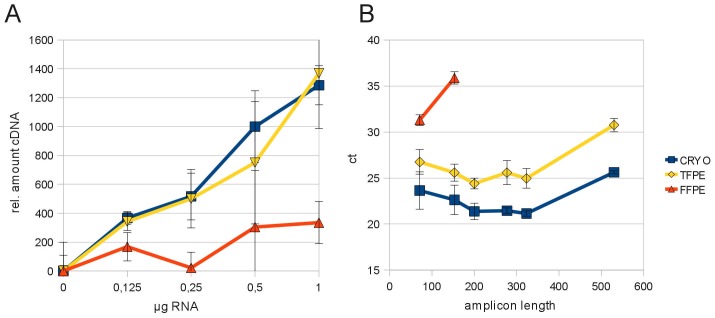
Impact of fixation on cDNA synthesis efficiency. (A) cDNA generation from RNA extracted from cryopreserved and TFPE human liver tissue is in direct correlation to the amount of template RNA. RNA extracted from FFPE tissue produces only small amounts of cDNA even when more template RNA is provided. cDNA from (A) was tested in the amplicon length qRT-PCR assay. (B) Ct values from the cryopreserved sample are in the range of 22–25cts. Ct values of TFPE samples are shifted by approximately 4 cycles but the appearance of the curve remains similar to that obtained from cryopreserved samples. In FFPE tissue the longer amplicons are not amplified, and even for the short amplicons the ct values are very high (31–36ct). Error bars in A depict standard deviation of the median qRT-PCR result in three individual cDNA preparations, or in B the standard deviation of three qRT-PCR analyses. RIN values and 260/280 ratios obtained from different samples: CRYO RIN: 7,7; 260/280: 1,97; TFPE RIN: 2,5; 260/280: 1,98; FFPE RIN: 2,7; 260/280: 2,01.

Aside the bias towards smaller RNA species introduced by fixation, we observed a generally lower efficiency of qRT-PCR using cDNA generated from RNA extracted from fixed samples as already shown in Figures 2 and 3. To elucidate whether this was a problem of PCR or of inefficient cDNA synthesis, we measured the amount of cDNA generated by reverse transcription of the same amount of input RNA extracted from corresponding frozen or fixed samples taken from a non-malignant human liver case. We determined the amount of cDNA generated in a reverse transcription reaction, after removal of the RNA from the cDNA/RNA hybrid molecules present in the cDNA sample by alkaline hydrolysis [27]. The concentration of the remaining cDNA was determined by fluorescence measurement using RiboGreen reagent. Under the assumption that cDNA generation was quantitative, a direct relationship between the amount of RNA introduced into the reaction and the amount of resulting cDNA would be expected. Using RNA from cryopreserved tissue this direct relationship was observed (Fig. 8A, blue line). However, RNA extracted from FFPE samples seemed to be an inferior template for cDNA generation as demonstrated by the inability to produce an equimolar amount of cDNA from a higher amount of RNA (Fig. 8A, red line). This indicates that cDNA synthesis is one of the most sensitive analytical steps which is impaired when using RNA from FFPE samples. Interestingly, RNA from samples fixed with the non-crosslinking fixative TTXMF was efficiently transcribed, comparable to RNA extracted from cryopreserved tissue (Fig. 8A, yellow line). Nevertheless, this clear advantage of RNA extracted from TFPE samples in cDNA synthesis did not fully translate into better results by qRT-PCR as there still was a substantial shift of ct values when compared to the results obtained from cryopreserved samples (Fig. 8B, blue and yellow line). However, by using the GAPDH amplicon length assay we found that cDNA obtained from TFPE samples was a better template for longer amplicons of the GAPDH assay which could be explained by the absence of RNA modifications as TTXMF is a non-crosslinking fixative. In this analysis of FFPE samples only the amplicons of 71 and 153 bp length were detected and the ct values were substantially higher (4–9 cycles) indicating inferior suitability for both cDNA synthesis and PCR amplification.

## Discussion

RNA expression analysis from FFPE tissues has been broadly used in biomedical research. However, several pre-analytical parameters like ischemia times, type of fixative, duration of fixation, storage and extraction techniques of biomolecules all impact on the quality of RNA extracted from fixed and paraffin embedded tissues. Several improvements to the standard FFPE protocol have been proposed to overcome this problem, such as fixation at low temperature or vacuum storage [31]. However, these approaches require special handling and changes in routine protocols. International initiatives for developing new pre-analytical technologies and tools for standardization of pre-analytical workflows of tissue-based molecular studies have been recently begun. One of the major activities in this field is SPIDIA (*Standardisation and improvement of generic pre-analytical tools and procedures for in-vitro diagnostics*, www.spidia.eu), a large-scale European collaborative project within 7^th^ EU framework program to investigate and standardize critical pre-analytical parameters of biological samples. In this project a new formalin-free, non-crosslinking tissue stabilisation technology (PAXgene Tissue) developed by PreAnalytiX (c/o Qiagen GmbH), has been evaluated. The PAXgene Tissue System resulted in high quality preservation of morphology, antigenicity, nucleic acids and phosphoproteins in paraffin-embedded tissue samples [32]–[34].

In addition, several groups have tested other possible alcohol-based substitutes for formalin in tissue fixation and could demonstrate improvements in preservation of nucleic acids and/or proteins [6]–[9], [12]. Among those alcohol-based fixatives TTXMF was found to be suitable for immunohistochemistry and fluorescence in-situ hybridisation of human breast cancer samples [35], [36]. While the preservation of histopathological features by the TTXMF fixative was deemed adequate (see also Figure S1), open questions remain mostly about safety issues, long term stability of samples and performance in complex stainings and immunohistochemistry. This is in line with conclusions drawn by Buesa in a comprehensive review of 27 formalin substitutes, including alcohol-based, non-alcohol-based fixatives and fixatives containing less than 10% formalin [37].

Despite several limitations for molecular diagnostics and toxicity concerns [38], we expect that FFPE will remain the gold standard for tissue based diagnostics in the near future because of the excellent preservation of morphology, the vast array of established protocols and last but not least the low costs. Thus, the question of how to reliably analyze RNA from FFPE blocks is and will remain of great interest and has been addressed in several previous publications. It is known that formalin fixation leads to substantial fragmentation of RNA molecules which impacts on qRT-PCR results [6]. Krafft et. al. [3] proposed the use of a reference mRNA (ACTB) to estimate the quality of RNA extracted from FFPE tissue blocks. The same gene was used by Penland et. al. [22] in a large study comparing the performance of RNA from 157 FFPE tumor samples in microarray hybridization experiments. Again, RNA quality was evaluated by qRT-PCR, as Bioanalyzer measurements were found to be unsuitable for RNA from formalin fixed samples. As quality control, the authors determined the delta ct ratio of the 3′ to the 5′ end of ACTB mRNA using TaqMan PCR. Since the data generated from RNA from FFPE samples clustered together in unsupervised cluster analysis of cDNA array expression data, a defined influence of fixation on mRNA species was discussed. The authors also discussed the possibility of using statistical tools to normalize for the variations introduced in microarray data by formalin fixation. In another detailed study by Koch et. al. [23], a standardized cell line model was used to determine the effects of formalin fixation on individual mRNA species. These experiments led to correction factors for individual genes which could be used to normalize the gene-to-gene variations. Sanchez-Navarro et. al. [24] used matched FFPE and fresh-frozen samples from human breast cancer to divide genes into groups based on the magnitude of the effect of fixation on qRT-PCR results of each individual gene. The authors recommended using only genes not affected by fixation for clinical molecular diagnostics.

These studies clearly demonstrate the need for improved quality indicators for RNA expression analysis from FFPE samples. Therefore, we have performed a comprehensive analysis of the pre-analytical workflow from tissue fixation to analysis of RNA by using qRT-PCR and cDNA array hybridization as readouts. Our studies aimed at identifying the most critical pre-analytical steps for molecular analysis of FFPE tissues that should be the target for improved quality control. We found that formalin and also TTXMF fixation followed by paraffin embedding led to substantial fragmentation of RNA resulting in very similar fragment length distribution in all samples regardless of their performance in downstream assays, an observation also made by other groups. The direct comparison of the qRT-PCR performance of corresponding CRYO and FFPE samples in a variety of tissues, and the correlation of these results to the RIN values of the template RNA showed that RIN values were a good quality indicator for RNA extracted from CRYO samples, but did not provide reliable information on the RNA quality of FFPE samples and subsequent performance in qRT-PCR assays. Furthermore we and others observed that occasionally RNA from formalin fixed samples shows high RIN values [39]. In these samples residual peaks of ribosomal RNA are present, albeit more diffuse and slightly shifted towards higher molecular weight. These residual peaks are still sufficient to generate high RIN values in the Agilent Bioanalyzer software but these samples did not demonstrate better performance in qRT-PCR assays thus again indicating the need for an alternative quality measurement for RNA extracted from fixed samples (Fig. 2). In addition to generally higher ct-values, qRT-PCR from FFPE samples was strongly influenced by amplicon length, demonstrating the need for assays with short amplicons for the analysis of FFPE tissues by qRT-PCR. However, RNA fragmentation did not make PCR amplification of amplicons longer than the average fragment size of the input RNA impossible, because different incomplete PCR products can anneal to each other leading to the assembly of the full length amplicon after several PCR cycles, which however results in higher ct values. It is thus important to choose a purification methodology which does not exclude small RNA molecules. Using an assay with a set of primers amplifying products of different length from the GAPDH gene, an approach also suggested by other groups [8], [11], [12], allowed accurate determination of the quality of RNA with regards to its performance in qRT-PCR assays. We found that increased fixation time in formalin did have a gradual detrimental influence on RNA quality, a difference that was not detectable by RIN measurement. RNA from FFPE or TFPE tissues also introduced profound and highly reproducible changes in the gene expression profiles generated by microarray analyses. However, the differences in gene expression detected by microarray analysis could not be reproduced reliably by qRT-PCR assays. Gene expression data was only consistent between microarrays and qRT-PCR when RNA from cryopreserved tissue was used.

Using a standardized qRT-PCR platform which detects 92 human cancer pathway related genes we also could show that there were profound variations in the gene-to-gene ratio introduced by fixation and paraffin embedding. These variations were even exacerbated by long-term storage. Interestingly, these gene-to-gene variations could not be overcome by using assays with short amplicon length, as all assays of this platform used amplicons smaller than 200 base pairs (Table S1). This different and gene-specific behavior of RNA in the context of sample pre-analytics could severely impact on the results of gene expression studies. This reinforces the observations made by other groups that formalin-fixation introduces constant, gene specific effects on qRT-PCR and microarray results [2], [4], [8], [12], [25].

RNA extracted from FFPE samples exhibited major gene to gene variation and a severe shift in ct values in qRT-PCR. As we do not routinely perform DNase pre-treatment of RNA extractions, contaminating DNA present in RNA preparations could interfere with quantification of RNA and subsequent qRT-PCR analysis of different samples. However, DNA contamination even up to 50% in the input amount for cDNA synthesis would only result in a ct-difference of one cycle in RT-PCR and can thus not explain the large differences seen in the amplicon length assays (Fig. 1, 2) and gene expression experiments. In addition the gene-to-gene variation seen in the qRT-PCR results obtained from the Human Molecular Mechanisms of Cancer TaqMan assay cannot be explained by different input amounts as these were measured from the same cDNA (Fig. 5).

Another aspect is the influence of formalin-fixation and paraffin-embedding on the efficiency of cDNA generation which we identified as the process which is most sensitive to formalin-induced pre-analytical effects. We found that RNA extracted from FFPE blocks was less efficiently transcribed to cDNA than RNA from TFPE or crypreserved samples. This inefficiency can interfere with many RNA based methods including next-generation-sequencing approaches, because most protocols require generation of cDNA templates before analysis. Changes to the cDNA generation protocol, like the addition of more MgCl_2_ [21] or using a higher random oligo concentration [20] have been proposed to tackle this issue. However, it is unclear if these modifications remove the gene-to-gene variations introduced by pre-analytical procedures.

In summary, we and others conclude that formalin fixation induces several detrimental changes in the RNA of human tissues. On the one hand, fixation and embedding leads to a breakdown of RNA molecules into small fragments, and on the other hand crosslinking fixatives like formalin modify RNA, leading to inefficient reverse transcription reactions. We have demonstrated a gene specific effect of fixation on individual mRNA species and suggest that impaired of cDNA generation could be the primary cause for this problem.

Furthermore, in the light of the multiple impacts of fixation on RNA we suggest that quality control of RNA from fixed tissues should not solely be assessed on electrophoretic RNA fragment length measurement or derived RIN values, but requires a multi-parameter approach. Depending on the level of standardization of the pre-analytical workflow we recommend testing RNA quality in addition to classical electrophoretic and spectrophotometric techniques by using a quantitative amplicon length assay and the cDNA synthesis efficiency measurement as presented here. Furthermore, improved comparability and reliability of RNA expression analysis of FFPE tissue samples has to be based on a combination of several quality assurance pillars, such as standardization of the pre-analytical workflow including warm and cold ischemia times, defined quality standards, multi-parameter quality control, and detailed sample associated information.

## Supporting Information

Figure S1 **Preservation of morphology in different malignant and non-malignant FFPE and TFPE tissues.** Hematoxylin and eosin staining of different human tissue samples fixed in parallel in neutral-buffered formaldehyde (FFPE) or Tissue Tek Xpress Molecular Fixative (TFPE). Corresponding FFPE and TFPE sections are shown. (A) Hyperplasia of the thyroid gland. (B) Renal cell carcinoma. (C) Liver adenoma. Original magnification (A–C): x 200.
(TIFF)

Table S1 **Amplicon sizes of the “Human molecular mechanisms of cancer” assay.**
(XLS)
