# Supplementary figures and images for: Quality Control of RNA Preservation and Extraction from Paraffin-Embedded Tissue: Implications for RT-PCR and Microarray Analysis
Source: PLoS One. 2013 Jul 31;8(7):e70714. doi: 10.1371/journal.pone.0070714 (PMC3729557; doi:10.1371/journal.pone.0070714)

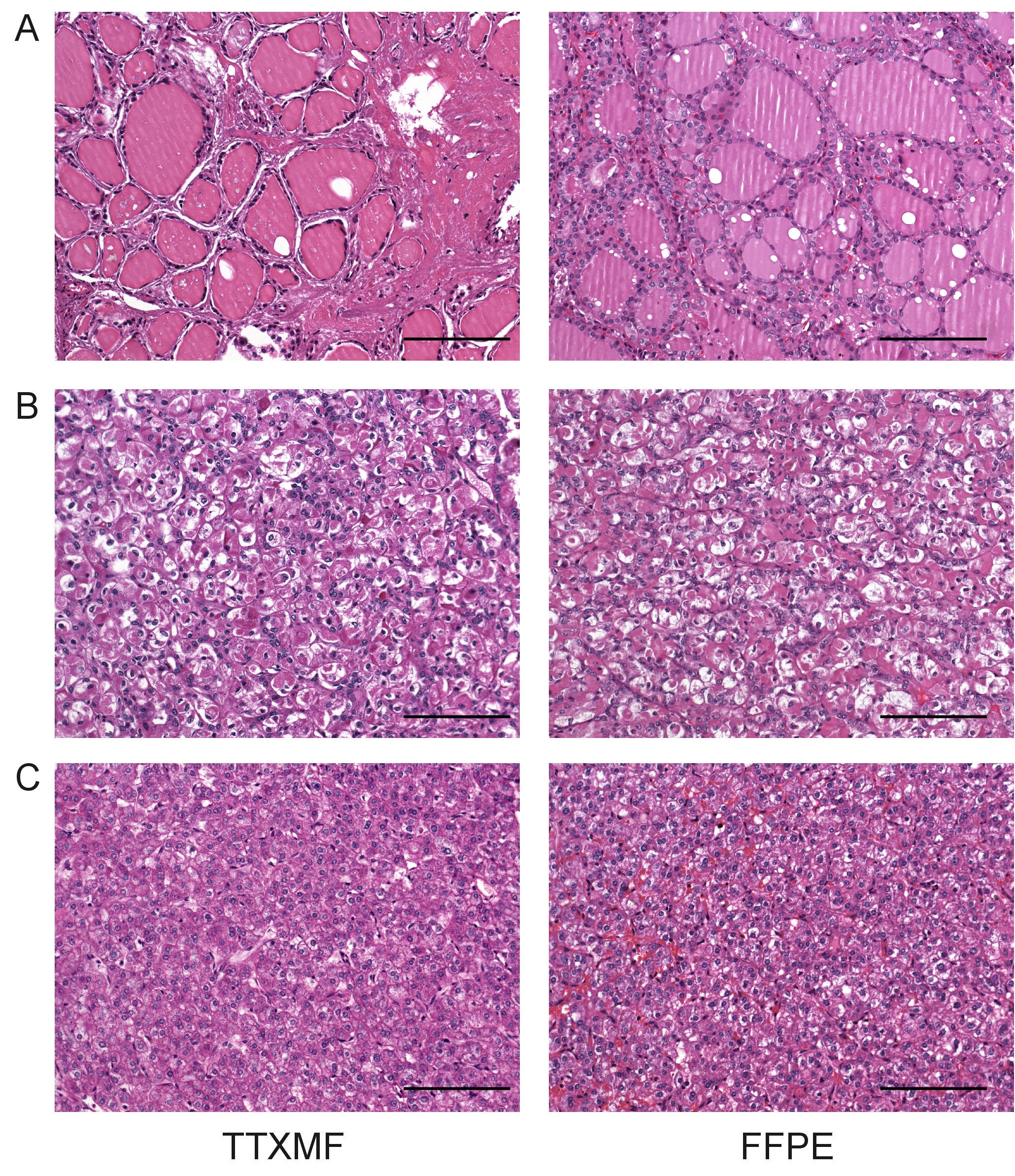

Supplement: Figure S1 — Preservation of morphology in different malignant and non-malignant FFPE and TFPE tissues. Hematoxylin and eosin staining of different human tissue samples fixed in parallel in neutral-buffered formaldehyde (FFPE) or Tissue Tek Xpress Molecular Fixative (TFPE). Corresponding FFPE and TFPE sections are shown. (A) Hyperplasia of the thyroid gland. (B) Renal cell carcinoma. (C) Liver adenoma. Original magnification (A–C): x 200. (TIFF) [file pone.0070714.s001.tif]
